# Relative prognostic value of TNM7 *vs* TNM6 in staging oesophageal cancer

**DOI:** 10.1038/bjc.2011.320

**Published:** 2011-08-16

**Authors:** T D Reid, L N Sanyaolu, D Chan, G T Williams, W G Lewis

**Affiliations:** 1 Department of Surgery, South East Wales Cancer Network, University Hospital of Wales, Cardiff, UK; 2 Department of Pathology, South East Wales Cancer Network, University Hospital of Wales, Cardiff, UK

**Keywords:** oesophageal cancer, TNM staging, prognosis

## Abstract

**Background:**

Stage migration consequent upon new cancer staging definitions may result in artifactual alterations in stage-specific survival and prognosis. The aim of this study was to determine the influence of the new TNM7 oesophageal cancer (OC) system on stage categorisation and survival when compared with historical controls.

**Methods:**

A total of 202 patients diagnosed with operable OC and undergoing oesophagectomy (118 neoadjuvant chemotherapy) were studied. Patients originally classified and staged using TNM6 were retrospectively re-staged using TNM7.

**Results:**

Re-classification of TNM7 resulted in stage migration in 11.9% of patients (9.9% downstaged, 2.0% upstaged) when compared with TNM6. Five-year survival for stages I, II and III was 78%, 46% and 18% using TNM6, compared with 62%, 51% and 18%, respectively, using TNM7. Univariable analysis revealed that histological grade (*P*=0.006), pT (*P*<0.0001), TNM6 pN (*P*<0.0001), TNM7 pN (*P*<0.0001), number of lymph node metastases (*P*<0.0001), TNM6 stage group (*P*<0.0001), TNM7 stage group (*P*<0.0001) and TNM7 prognostic group (*P*<0.0001) were all associated with survival. Multivariable analysis revealed that only the TNM7 prognostic group was independently and significantly associated with survival.

**Conclusion:**

TNM7 is a better prognostic tool than TNM6 and represents an important advance in staging OC.

The Tumour, Nodes, Metastases (TNM) Classification system, published by the International Union Against Cancer, is the gold standard cancer staging system used worldwide (Sobin *et al*, 2009). Its objectives include assisting in the planning of therapy, informing prognosis, allowing evaluation of results and facilitating information exchange between treatment centres, and is updated periodically to incorporate evidence-based evolution (Sobin *et al*, 2009). However, the 2002 TNM sixth Edition (TNM6) has been considered to be a tool of limited prognostic value in oesophageal cancer (OC), mainly because lymph node (N) stage was limited by definition as a binary variable (N0 or N1), regardless of the actual lymph node metastasis count (Sobin and Wittekind, 2002).

The recently published TNM seventh edition (TNM7) replaced TNM6 with effect from 2010, and incorporated major modifications with regard to OC, in particular related to the individual T, N and M stage criteria, and stage groups (Sobin *et al*, 2009). Moreover, TNM7 introduced a new system of prognostic groups in which other prognostic variables are combined with T, N and M categories for stage I and II tumours, but not for stage III and IV tumours, where the prognostic grouping depends entirely on traditional T, N and M categories. TNM7 prognostic groupings also differ with regard to histopathological cell type. Prognostic grouping for both adenocarcinoma (ACA) and squamous cell carcinoma (SCC) takes account of tumour grade for stage I and II tumours, but for SCC, the anatomical site within the thoracic oesophagus is also incorporated for stage I and II tumours (Sobin *et al*, 2009). However, the principal TNM7 upgrade relates to the classification of lymph node stage, which has a major influence on defining specific stage groups. The number of lymph node metastases has long been considered to be the key and defining prognostic factor for patients diagnosed with OC (Kawahara *et al*, 1998), and in addressing this issue TNM7 reclassifies lymph node positive tumours into four groups (N0-3) based on the relative burden of nodal metastases (Sobin *et al*, 2009). These groups are defined as N0, N1 (1–2 lymph nodes), N2 (3–6 lymph nodes) and N3 (>6 lymph nodes). Stage groups have also been revised and expanded to account for this modified N stage.

The aims of this study therefore, were to determine the influence of the new TNM7 staging system on OC histopathological stage categorisation and related survival, when compared with historical control data derived with TNM6, and to determine the relative accuracy of TNM6 and TNM7 in predicting prognosis. The setting was a regional upper gastrointestinal cancer network in South Wales serving a population of 1.3 million.

## Materials and methods

Consecutive patients who underwent potentially curative surgery for OC by a regional upper GI cancer network were identified from a prospectively maintained database. Patients were excluded if they had a complete pathological response to neoadjuvant treatment, involved longitudinal resection margins, high-grade dysplasia in the absence of invasive malignancy or if complete histological information on the numbers of involved lymph nodes was missing. Complete pathological data were available on 202 patients, all of whom underwent oesophagectomy between 1998 and 2010. The median age of the patients was 61 (range 35 to 79) years. There were 161 (79.7%) males and 41 (20.3%) females. A total of 169 patients had ACAs (83.7%) and 33 patients had SCCs (16.3%).

Preoperative staging involved computed tomography and endoluminal ultrasonography, and was in accordance with TNM6 definitions. All patients were discussed at a regional specialist multi-disciplinary team meeting with management plans individually tailored according to factors relating to both cormobidity and tumour stage. In general, fit patients with tumours of stage T3 and equivocal T4, N0 and N1 were considered for neoadjuvant therapy before surgery. Less fit patients and those with T1-2, N0 disease were considered for surgery alone. A total of 121 patients underwent standard subtotal oesophagectomy as described by Lewis (1946) and Tanner (1947). Transhiatal resection, as described by Orringer (1985) was performed in 81 patients. This was employed selectively in patients with ACC of the lower third of the oesophagus who had significant cardiorespiratory co-morbidity. Neoadjuvant chemotherapy or chemoradiotherapy were given to 87 patients and 31 patients, respectively.

All patients were originally staged histopathologically in accordance with TNM6, and then retrospectively re-staged using TNM7. The primary outcome measure was survival. Clinical follow-up was 3 monthly intervals for the first year following surgery, decreasing to 6 monthly intervals thereafter for 5 years or until death. A total of 187 patients (92.6%) were followed-up for 5 years or until death. Death certification was obtained from the Office for National Statistics.

The regional ethics committee were contacted regarding this study, but a formal application was deemed unnecessary.

### Statistical analysis

Data were expressed as median (range). Methods appropriate for non-parametric data were used. Cumulative survival was calculated according to the life-table method of Kaplan and Meier (1958), and differences in survival between groups of patients were analysed with the log-rank test. Multivariable analysis of factors influencing survival was performed using Cox's proportional hazards model (Cox, 1972). The gradient of Schoenfeld (partial) residuals (Schoenfeld, 1982) *vs* time was calculated using linear regression, for each of the variables entered into the multivariable model, in order to test for violations of the proportional hazards assumption. Data analysis was performed using SPSS version 18.0 (Chicago, IL, USA).

## Results

The T and N stages, stage groups and prognostic groups of the patients are shown in Table 1, and the proportion of patients who migrated between stages when TNM7 was applied are summarised in Table 2. There were no changes observed in OC pT stage, but significant changes were observed in pN stage. Of the 110 patients (54.5%) with lymph node metastases classified as pN1 by TNM6, 56 (27.7%) remained pN1, 35 (17.3%) were re-classified as pN2 and 19 (9.4%) patients were re-classified as pN3 by TNM7. With regard to stage groups, the number of patients with stage I disease almost doubled under TNM7, whereas in contrast the number of patients with stage II tumours was reduced by almost a third. The number of patients with stage III tumours increased slightly when classified by TNM7, whereas the number of patients with stage IV tumours remained unchanged. Downstage migration occurred in 20 (9.9%) patients when classified by TNM7 (stage II to I). Upstage migration occurred in 4 (2.0%) patients (stage II to III). When TNM7 prognostic group allocations were compared with TNM6 allocations, fewer were classified as stage I, and more were classified as stage II. The numbers of patients with stages III and IV tumours remained unchanged. Eleven patients from the early period of this series could not be allocated a prognostic group because of pathology reports that failed to comment on tumour grade.

### Survival

**Figure 1:**
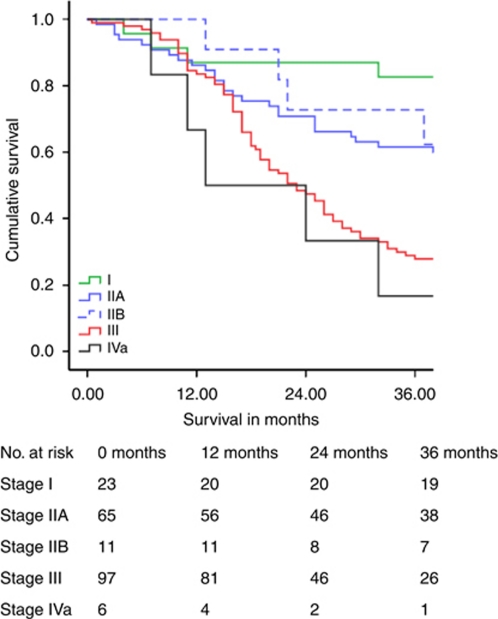
Oesophageal cancer survival related to TNM6 stage groups.

**Figure 2:**
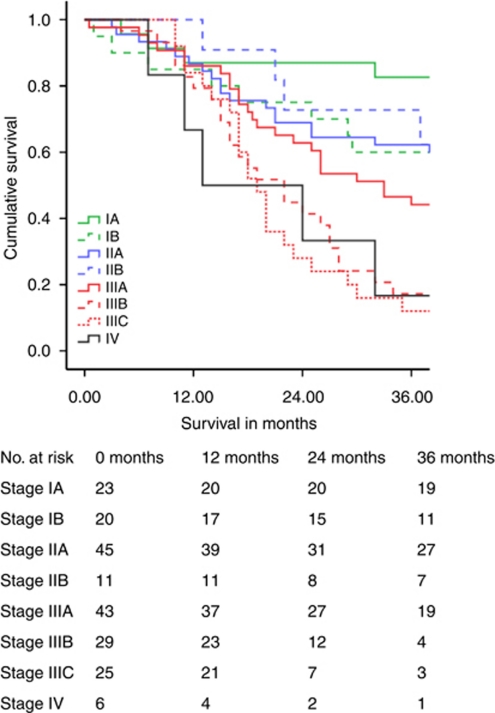
Oesophageal cancer survival related to TNM7 stage groups.

**Figure 3:**
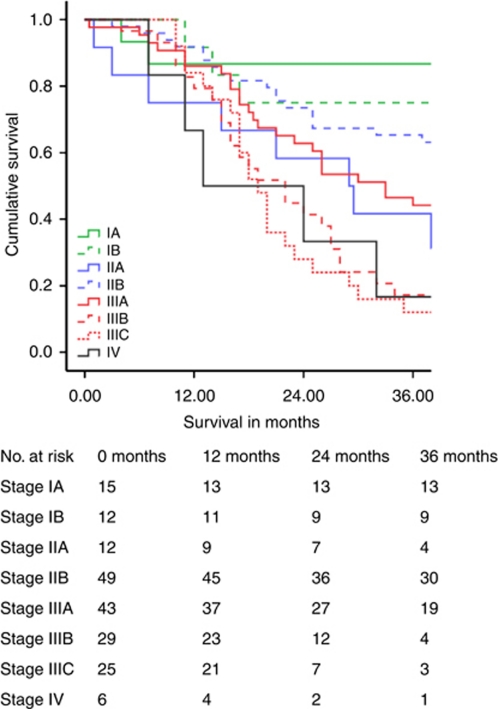
Oesophageal cancer survival related to TNM7 prognostic groups.

Table 3 illustrates cumulative 5-year survival related to the stage. Survival related to TNM7 is shown separately. Figures 1, 2, 3 demonstrate Kaplan–Meier survival curves for OC by TNM6 and TNM7 stage groups, and TNM7 prognostic groups.

For stage I OC, 5-year survival by TNM7 was poorer by 16.1% when compared with TNM6. In contrast stage II OC 5-year survival by TNM7 improved by 4.4%, and median survival improved by 17 months. Survival for patients with stage III and IV tumours remained the same. Allocation of TNM7 prognostic groups produced survival plots that were midway between those obtained with TNM6 and TNM7 stage groups for patients with stage I and II tumours, and were unchanged for patients with stage III and IV tumours.

Univariable analysis of the factors associated with survival is shown in Table 4. On multivariable analysis including the following variables: – tumour grade, pT stage, TNM6 pN stage, TNM7 pN stage, TNM6 stage group, TNM7 stage group, TNM7 prognostic group and the number of lymph node metastases – only the TNM7 prognostic group emerged as significantly and independently associated with survival (Table 5).

Further univariable and multivariable sub analyses, related to histopathogical cell type, of the factors associated with survival in the cohort of 169 ACA patients revealed an identical picture. The cohort of 33 SCC patients was considered too small to perform any meaningful survival analysis.

No significant violations of the proportional hazards assumption were identified by means of the gradient of Schoenfeld (partial) residuals *vs* time.

## Discussion

This represents the only Western study to date to compare the effects of the new TNM7 staging system to TNM6 for OC. The principal findings were that stage migration occurred in 11.9% of patients, and this resulted in significant artifactual change in survival rates for early stage disease (I and II), but no change in outcome for more advanced stage disease (III and IV). Prognosis was better predicted by TNM7 when compared with TNM6, and specifically by the new prognostic groups incorporated in TNM7. This datum therefore provides strong support for the updated TNM7 staging system for OC, and lends further weight to the validity of the data-driven approach used to derive this radical update (Rice *et al*, 2010).

The study has several strengths. The patient numbers are relatively large by Western standards, and represent a consecutive series treated by a single UK cancer network. All patients received stage-directed treatment by a specialist regional multidisciplinary team with considerable experience in the treatment of oesophagogastric cancer. The surgery was performed by specialist upper GI surgeons whose results have been well audited (Morgan *et al*, 2009) and shown to be equivalent or better than those reported in the UK-based MRC OEO2 randomised trial (MRC Oesophageal Cancer Working Party, 2002; Morgan *et al*, 2009). The patients resided in a well-defined geographical area, and the follow-up data is especially robust with dates and causes of death obtained from the Office of National Statistics.

Nevertheless, there are potential limitations. Although the numbers of patients were large for a single UK region, they are relatively small when compared with the large multicentre study undertaken to derive TNM7 for OC (Rice *et al*, 2010). However Rice *et al* describe a patient series who underwent surgery alone, whereas our patients were treated in line with current UK practice, where neoadjuvant chemotherapy is the standard of care for locoregionally advanced OC (MRC Oesophageal Cancer Working Party, 2002). In addition, total lymph node harvests were variable, with a median of 11 nodes (range 1–38) retrieved. We have reported previously that the prognosis of surgically resected OC is highly dependent on the numbers of lymph nodes examined pathologically, again arguably due to stage migration effects (Twine *et al*, 2009). Hsu *et al* from Taiwan have recently reported a comparison of TNM6 *vs* TNM7 in surgically resected oesophageal SCC. Their findings confirmed the predictive value of the new pN and pM stage criteria and they concluded that TNM7 constituted an improvement over TNM6 in terms of informing outcome (Hsu *et al*, 2010). However, although their sample size was large (392), as might be expected from its geographical origin, this study consisted exclusively of patients diagnosed with squamous cell cancer, and it is therefore uncertain how applicable their conclusions are to patients diagnosed with ACA, the predominant Western tumour, which accounts for the vast majority (83.7%) of patients in this study. The cohort of patients diagnosed with SCC in this study was too small for valid comparison with the findings of Hsu *et al.*

Improving the accuracy of any given staging system represents a fundamental aim of modifying and upgrading prognostic models, and TNM7 appears to have been successful in this regard related to OC. Such a benefit must, however, be balanced against potential and inherent disadvantages of modifying histopathological staging. The ‘Will Rogers Phenomenon’ is a perceived paradox named after a quote attributed to the US comedian and social commentator Will Rogers (1879–1935). Referring to migration during the American economic depression of the 1930s, he allegedly said

‘When the Okies left Oklahoma and moved to California, they raised the average intelligence level in both states’

An analogous phenomenon is the concept of cancer stage migration, whereby changes in staging result in apparent differences in group outcomes, yet individual patient outcomes remain unchanged. The first report of this phenomenon described a cohort of lung cancer patients that had artifactually better stage-specific survival than historical controls from the same institution (Feinstein *et al*, 1985). The difference was explained by the combination of both a prognostically favourable lead-time bias and stage migration, as a result of more advanced imaging techniques (Feinstein *et al*, 1985). Changes in the rules governing the grading of prostate cancer biopsies, and improved histopathological processing of bladder cancer specimens, have produced similar stage migration effects in urological oncology (Gofrit *et al*, 2008). In this study, significant yet purely artifactual changes in the survival of early-stage OC have been demonstrated, through the application of the TNM7 system, for the same reasons. Such changes may not only be mistakenly attributed to the effects of treatment, but also equally importantly risk preventing meaningful comparison of patient outcomes with historical controls, including those published in clinical trials. Indeed, our study indicates that it is now imperative for the results of clinical trials to be reported in relation to the TNM stage classification used. There is also a responsibility upon the clinicians involved in revising and updating the TNM staging system to ensure that modifications are based on the best available evidence, using pathological criteria that are known to be reproducible, to justify any concomitant problems that an update may bring. In the case of the colorectal-cancer TNM staging system, major concerns have been raised within the pathology community regarding modifications incorporated within TNM6 and TNM7 because of a perceived lack of a sound evidence base (Quirke *et al*, 2007; Quirke *et al*, 2010) and because of this, UK colorectal-cancer staging continues to be classified by means of TNM5, more than a decade after its publication (Quirke *et al*, 2007)

In conclusion, TNM7 is a better prognostic tool than TNM6 for OC, predominantly due to the inclusion of the relative burden of the lymph node metastases count. Moreover, the new system of prognostic grouping in TNM7, taking account of tumour grade and site, has further refined staging accuracy when compared with the traditional anatomically based stage grouping. TNM7 should therefore also form the basis for the radiological reporting and staging of all modalities for OC. Notwithstanding the significant benefit TNM7 provides, clinicians must be aware of the Will Rogers Phenomenon in the context of cancer staging, and avoid drawing misleading conclusions when comparing current patient outcomes with those of historical controls.

## Figures and Tables

**Table 1:** Details of the patients' staging by TNM6 and TNM7

	**TNM6**	**TNM7**
*T stage*		
T1	29 (14.4)	29 (14.4)
T2	29 (14.4)	29 (14.4)
T3	130 (64.4)	130 (64.4)
T4	14 (6.9)	14 (6.9)
		
*N stage*		
N0	92 (45.5)	92 (45.5)
N1	110 (54.5)	56 (27.7)
N2	N/A	35 (17.3)
N3	N/A	19 (9.4)
		
*Stage groupings*		
I	23 (11.4)	43 (21.3)
II	80 (39.6)	56 (27.7)
III	93 (46.0)	97 (48.0)
IV	6 (3.0)	6 (3.0)
		
*Prognostic groupings*		
I	N/A	27 (13.4)
II	N/A	61 (30.2)
III	N/A	97 (48.0)
IV	N/A	6 (3.0)
Not determined	N/A	11 (5.4)

Abbreviations: N/A=not applicable; TNM=tumour, nodes, metastases.

Figures are number of patients (%).

**Table 2:**
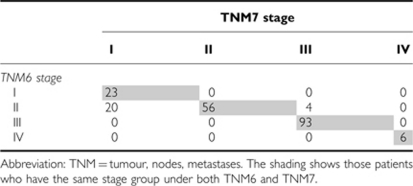
Stage migration related to TNM7 classification

**Table 3:** Stage-by-stage patient survival

**Survival**	**TNM6 stage groups**	**TNM7 stage groups**	**TNM7 prognostic groups**
*Stage I*			
Median (months)	N/A	111	111
5 year (%)	78.3	62.2	67.7
			
*Stage II*			
Median (months)	47	64	48
5 year (%)	46.3	50.7	48.6
			
*Stage III*			
Median (months)	23	23	23
5 year (%)	18.3	17.6	17.6
			
*Stage IV*			
Median (months)	13	13	13
5 year (%)	0	0	0

Abbreviations: N/A=not applicable; TNM=tumour, nodes, metastases.

**Table 4:** Univariable analysis of factors influencing survival

**Variable**	***χ* ^2^**	**DF**	***P*-value**
Age	48.020	41	0.210
Gender	1.039	1	0.308
Histological cell type	2.250	2	0.308
Histological tumour grade	10.260	2	0.006
Operative approach (TT *vs* TH)	0.795	1	0.373
Neoadjuvant therapy	0.627	1	0.429
T stage (same in TNM6 and TNM7)	21.514	3	<0.0001
N stage (TNM6)	21.499	1	<0.0001
N stage (TNM7)	37.509	3	<0.0001
Number of lymph node metastases	61.677	12	<0.0001
Stage groupings (TNM6)	36.587	4	<0.0001
Stage groupings (TNM7)	50.531	7	<0.0001
Prognostic groupings (TNM7)	47.147	7	<0.0001

Abbreviations: DF=degrees of freedom; TH=trans hiatal; TNM=tumour, nodes, metastases; TT=trans thoracic.

**Table 5:** Multivariable analysis of factors influencing oesophageal cancer survival

**TNM7 prognostic stage**	**Hazard ratio**	**95% CI**		***P*-value**
Stage IA	Reference group			
Stage IB	3.901	1.034	14.721	0.045
Stage IIA	5.994	1.586	22.659	0.008
Stage IIB	4.346	1.303	14.503	0.017
Stage IIIA	5.734	1.743	18.869	0.004
Stage IIIB	10.838	3.244	36.211	<0.001
Stage IIIC	13.130	3.873	44.511	<0.001
Stage IV	11.565	2.743	48.760	<0.001

Abbreviations: CI=confidence interval; TNM=tumour, nodes, metastases.

## References

[bib1] Cox DR (1972) Regression models and life tables. J R Stat Soc B:34(2): 187–200

[bib2] Feinstein AR, Sosin DM, Wells CK (1985) The Will Rogers phenomenon. Stage migration and new diagnostic techniques as a source of misleading statistics for survival in cancer. N Engl J Med 312: 1604–1608 4000199 10.1056/NEJM198506203122504

[bib3] Gofrit ON, Zorn KC, Steinberg GD, Zagaja GP, Shalhav AL (2008) The Will Rogers phenomenon in urological oncology. J Urol 179(1): 28–33 17997434 10.1016/j.juro.2007.08.125

[bib4] Hsu P-K, Wu Y-C, Chou T-Y, Huang C-S, Hsu W-H (2010) Comparision of the 6^th^ and 7^th^ editions of the American Joint Committee on Cancer Tumour-Nodes-Metastasis staging system in patients with resected esophageal carcinoma. Ann Thorac Surg 89: 1024–1031 20338302 10.1016/j.athoracsur.2010.01.017

[bib5] Kaplan EL, Meier P (1958) Non-parametric estimation from incomplete observations. J Am Stat Assoc 58: 457–481

[bib6] Kawahara K, Maekawa T, Okabayashi K, Shiraishi T, Yoshinaga Y, Yoneda S, Hideshima T, Shirakusa T (1998) The number of lymph node metastases influences survival in esophageal cancer. J Surg Oncol 67(3): 160–163 9530885 10.1002/(sici)1096-9098(199803)67:3<160::aid-jso3>3.0.co;2-7

[bib7] Lewis I (1946) The surgical treatment of carcinoma of the oesophagus, with special reference to new operation for growths of middle third. Br J Surg 34: 18–31 20994128 10.1002/bjs.18003413304

[bib8] Medical Research Council Oesophageal Cancer Working Group (2002) Surgical resection with or without preoperative chemotherapy in oesophageal cancer: a randomised controlled trial. Lancet 359: 1727–1733 12049861 10.1016/S0140-6736(02)08651-8

[bib9] Morgan MA, Lewis WG, Casbard A, Roberts SA, Adams R, Clark GW, Havard TJ, Crosby TD (2009) Stage-for-stage comparision of definitive chemoradiotherapy, surgery alone and neoadjuvant chemotherapy for oesophageal carcinoma. Br J Surg 96: 1300–1307 19847875 10.1002/bjs.6705

[bib10] Orringer MB (1985) Transhiatal esophagectomy for benign disease. J Thorac Cardiovasc Surg 90: 649–655 4058037

[bib11] Quirke P, Cuvelier C, Ensari A, Glimelius B, Laurberg S, Ortiz H, Piard F, Punt CJA, Glenthoj A, Pennickx F, Seymour M, Valentini V, Williams G, Nagtegaal I (2010) Evidence-based medicine: the time has come to set standards for staging. J Pathol 221: 357–360 20593493 10.1002/path.2720

[bib12] Quirke P, Williams GT, Ectors N, Ensari A, Piard F, Nagtegaal I (2007) The future of the TNM staging system in colorectal cancer: time for a debate? Lancet Oncol 8: 651–657 17613427 10.1016/S1470-2045(07)70205-X

[bib13] Rice TW, Rusch VW, Ishwaran H, Blackstone EH (2010) Cancer of the esophagus and esophagogastric junction. Data-driven staging for the seventh edition of the American Joint Committee on Cancer/International Union Against Cancer Staging Manuals. Cancer 116: 3763–3773 20564099 10.1002/cncr.25146

[bib14] Schoenfeld D (1982) Partial residuals for the proportional hazards regression model. Biometrika 69(1): 239–241

[bib15] Sobin LH, Gospodarowicz MK, Wittekind C (eds) (2009) UICC TNM Classification of Malignant Tumours, 7th edn. Wiley: New York

[bib16] Sobin LH, Wittekind C (eds) (2002) UICC TNM Classification of Malignant Tumours, 6th edn. Wiley: New York

[bib17] Tanner IC (1947) The present position of carcinoma of the oesophagus. Postgrad Med J 23: 109–139 20287301 10.1136/pgmj.23.257.109 PMC2529504

[bib18] Twine CP, Lewis WG, Morgan MA, Chan D, Clark GW, Havard T, Crosby TD, Roberts SA, Williams GT (2009) The assessment of prognosis of surgically resected oesophageal cancer is depended on the number of lymph nodes examined pathologically. Histopathology 55: 46–52 19614766 10.1111/j.1365-2559.2009.03332.x

